# Improving Outcomes in Cancer Patients on Oral Anti-Cancer Medications Using a Novel Mobile Phone-Based Intervention: Study Design of a Randomized Controlled Trial

**DOI:** 10.2196/resprot.4041

**Published:** 2014-12-23

**Authors:** Stephen Agboola, Clare Flanagan, Meghan Searl, Aymen Elfiky, Joseph Kvedar, Kamal Jethwani

**Affiliations:** ^1^Partners Healthcare Center for Connected HealthBoston, MAUnited States; ^2^Massachusetts General HospitalBoston, MAUnited States; ^3^Harvard Medical SchoolBoston, MAUnited States; ^4^Dana Faber Cancer InstituteBoston, MAUnited States

**Keywords:** cancer, oral anti-cancer medication, mobile application, randomized controlled trial, self-care, mHealth, medication adherence

## Abstract

**Background:**

The widespread and increasing use of oral anti-cancer medications has been ushered in by a rapidly increasing understanding of cancer pathophysiology. Furthermore, their popular ease of administration and potential cost savings has highlighted their central position in the health care system as a whole. These facts have heightened appreciation of the unique challenges associated with the use of oral anti-cancer medications; especially in the long-term use of these medications and the associated side effects that may impede optimal adherence to their use. Therefore, we developed ChemOtheRapy Assistant, CORA, a personalized mobile phone–based self-management application to help cancer patients on oral anti-cancer medications.

**Objective:**

Our objective is to evaluate the effect of CORA on adherence to oral anti-cancer medications and other clinically relevant outcomes in the management of patients with renal and prostate cancer.

**Methods:**

The study will be implemented as a 2-parallel group randomized controlled trial in 104 patients with renal or prostate cancer on oral anti-cancer medications over a 3-month study period. The intervention group will use CORA in addition to usual care for self-management while the control group will continue care as usual. Medication adherence will be measured objectively by a Medication Event Monitoring System device and is defined as the percentage of prescribed doses taken. We will also assess the effect of the intervention on cancer-related symptoms measured by the MD Anderson Symptom Inventory and unplanned hospital utilizations. Other outcomes that will be measured at study start, midpoint, and endpoint are health-related quality of life, cancer-related fatigue, and anxiety. Group differences in medication adherence will be examined by t tests or by non-parametric Mann-Whitney tests if the data are not normally distributed. Logistic regression will be used to identify potential predictors of adherence.

**Results:**

We expect to have results for this study before the end of 2016.

**Conclusions:**

This novel mobile phone–enabled, multimodal self-management and educational intervention could lead to improvements in clinical outcomes and serve as a foundation for future mHealth research in improving outcomes for patients on oral anti-cancer medications.

##  Introduction

Oral anti-cancer medications (OAMs) are increasingly being used as an alternative to traditional intravenous chemotherapy in cancer management [1]. Factors promoting this new trend in cancer management include increased survival times requiring long-term therapy, favorable acceptability of these newer medications among patients, convenience and ease of administration, and potential cost savings due to less time spent in the hospital [2,3]. However, this shift significantly increases the burden of self-care for patients similar to that in many chronic diseases where compliance, defined as “implementation by the patient of the therapeutic plan that has been established”, is the direct responsibility of the patients or caregivers [3]. Medication adherence in cancer therapy has significant impact on treatment efficacy and development of toxicities [4]. Studies have reported variable medication adherence rates in cancer patients on OAM regimens, with adherence rates ranging from less than 20% to 100% [5,6]. Suboptimal adherence not only leads to loss of treatment efficacy and increased toxicity, but it also results in increased hospital utilization, longer hospital stays, and increased health care costs [6,7].

An important component of self-managing these medications is managing the adverse effects, which are not altogether different from their intravenous counterparts, resulting from taking these powerful medications. These adverse effects threaten their continued use, and most commonly result in non-adherence to the recommended treatment plan [8]. In addition to the drug-related adverse effects, other barriers to optimal adherence include inadequate treatment supervision, poor communication with providers, and other patient-related barriers like poor knowledge, self-efficacy, and motivation [9]. Multidisciplinary approaches to home-based management of chemotherapy by oncology nurses, physicians, and health educators as well as some pharmacy-based care management programs have shown some success in addressing this problem [10-12]. However, these approaches are not scalable unless they are made more cost-effective. We hypothesize that creatively leveraging mobile technology could potentially mimic the effect of such intensive and coordinated interventions to improve outcomes.

Therefore, we developed ChemOtheRapy Assistant, CORA, a personalized mobile-based, multimodal self-management intervention designed for extensive patient education and symptom management, based on evidence from clinical guidelines and direct patient/caregiver stakeholder feedback, to improve medication adherence in cancer patients on OAMs. We expect that this intervention, delivered through a mobile device, compatible with both iOS and Android devices, will forge engagement to improve outcomes similar to those seen in chronic disease management programs [13-15]. This novel project is being implemented in a collaborative effort by a multidisciplinary team of oncology physicians, nurses, pharmacists, and psychologists.

For this study, we will focus on patients with renal cell cancer (RCC) and prostate cancers. These specific cancers accounts for significant morbidity and mortality due to cancer in the United States [16]. OAMs are either first-line therapies (eg, for RCC) or very commonly used at some point in management of these cancers; and with increasing survival data, there is evidence for their use on an extended basis. We will be including patients with RCC or prostate cancer on any of the following four oral VEGF inhibitors—sorafenib, sunitinib, pazopanib, axitinib—or everolimus, an mTOR inhibitor. These OAMs are commonly used in patients with metastatic renal cell carcinoma with common side effects including hypertension, diarrhea, nausea, and hand-foot syndrome [17]. We hypothesize that patients with RCC and prostate cancers on OAMs who use CORA will develop increased competency for self-care to manage side effects associated with their medications, which will translate to higher medication adherence and improved clinical outcomes compared to cancer patients on OAMs who do not use the mobile-based intervention.

Our primary aim is to assess the effect of CORA on adherence to OAMs. We define medication adherence as the percentage of prescribed doses taken.

For our secondary aims, we will be assessing the effect of CORA on the severity of symptoms, unplanned hospital utilizations, health-related quality of life, cancer-related fatigue, and anxiety in patients on OAMs. Additionally, we will also assess usability and participant satisfaction with the app.

## Methods

### Trial Design

**Figure 1:**
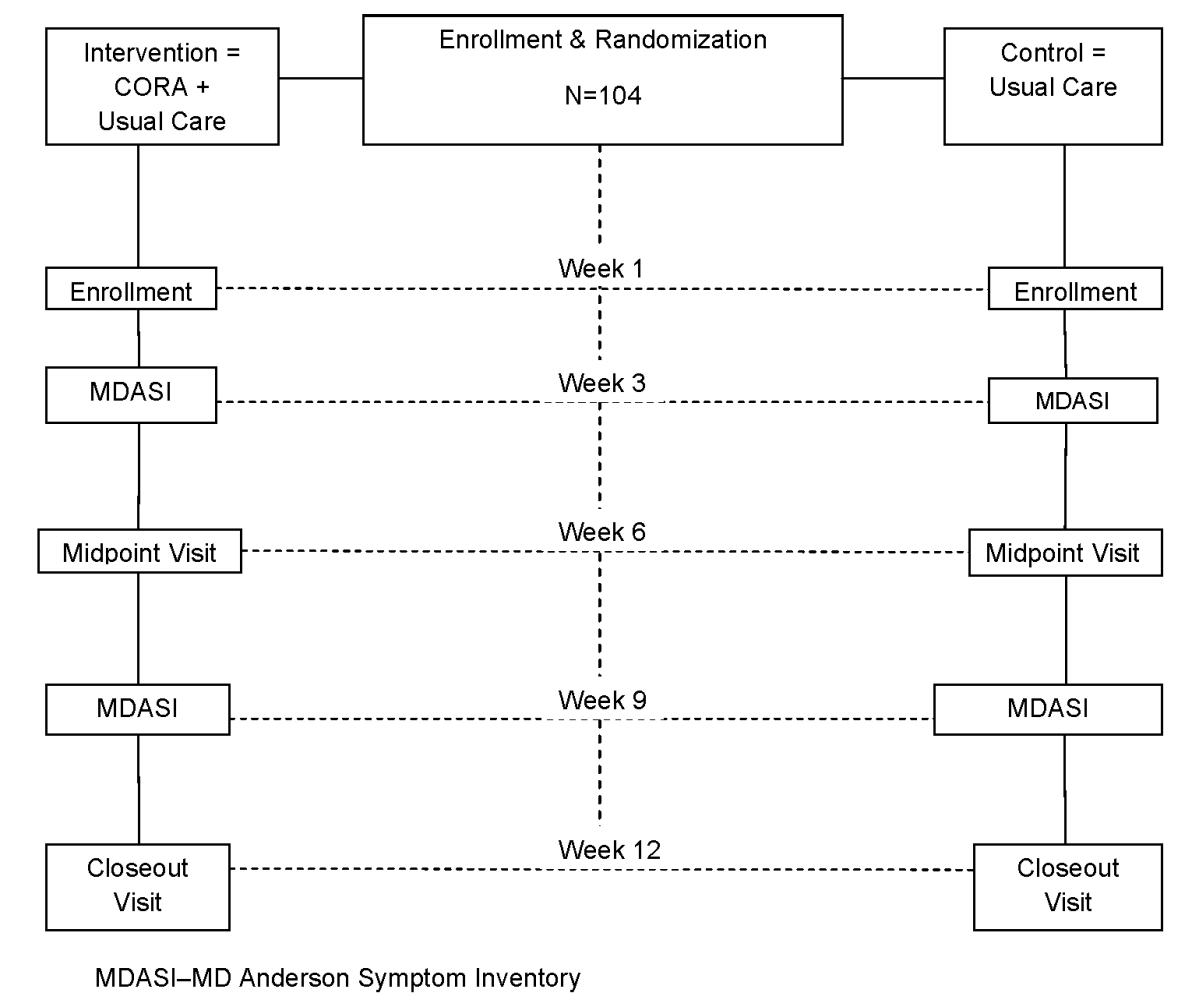
Schematic summary of the trial design.

This study will be implemented as a 2-parallel group randomized controlled trial, intervention versus usual care, with multiple assessments over a 3-month follow-up period. Figure 1 shows the research design.

### Participant Inclusion/Exclusion Criteria

Participants for this study will be recruited from the Dana Faber Cancer Institute (DFCI), Boston, Massachusetts. To be considered eligible to participate in the study, patients must fulfill all eligibility requirements: patients must be ambulatory, age 18 years or older, able to consent for self, have a diagnosis of renal or prostate cancer, commencing a new cycle of OAMs, and able to read and speak English. In addition, since the intervention will be deployed on a mobile phone, they must have an Apple or Android mobile phone and be willing to download the app on their mobile phone to use the intervention.

Exclusion criteria include life expectancy less than 3 months as determined by the managing oncologist, current participation in a similar study geared at improving medication adherence or in investigational drug trials where adverse effects have not been fully elucidated, and presence of significant psychiatric comorbidities and memory or cognitive impairments. A significant psychiatric condition includes any condition that creates major distress for a patient or markedly impairs the patient’s daily functioning. This includes, but is not limited to, acute psychoses, major depressive disorder, dementia, etc.

### Recruitment Procedure

Participants for this trial will be drawn from cancer patients receiving care at the DFCI on OAMs. On a weekly basis, the research team will identify patients being started on a new cycle of OAM from chart reviews. After identifying potentially eligible patients, we will ask the managing oncologists to give approval to contact the patient about participating in the study. Thereafter, recruitment letters will be sent to all approved patients. One week after recruitment letters have been mailed, research assistants will attempt to contact subjects by phone to provide further detail regarding the study. If subjects are interested in participating, study staff will schedule an enrollment visit with subjects at the DFCI at the subject’s convenience.

All enrollment procedures, including the informed consent process, completing enrollment surveys, and randomization procedures will be performed by trained research assistants. At the enrollment visit, all participants will be instructed to continue to receive usual medical care from their physicians as usual. They will also receive a Medication Event Monitoring System (MEMS) device, a valid measure of adherence used in many chronic diseases, to monitor medication adherence in this study. The bottle cap has a microprocessor that records all instances and times that the bottle is opened. They will be instructed to open their pill bottles only when they want to take their medications. Only subjects randomized to the intervention arm will download and be able to use the CORA mobile app.

### Intervention

#### Framework for Intervention

This mobile-based multimodal self-management program is based on integrated evidence from the American Society of Clinical Oncology and the Oncology Nursing Society standards for safe chemotherapy administration clinical guidelines, the medical literature, and experiences from clinical practice. Our goal is to increase adherence to OAMs by empowering patients to better manage their medications and associated side effects. The focus will be on helping patients develop competency in preventing, or the early identification of, adverse effects that may impact adherence and hence negatively impact clinical outcomes. Our approach is grounded in extensive education and symptom management. Our strategies are as described below.

#### Coaching to Improve Self-Efficacy for Self-Care

Prior to the onset of oral chemotherapy, intensive education (60-90 minutes in duration) is usually done in the hospital at a time when patients are anxious and distracted by the high volume of information being directed at them. Additional educational sessions are also done on a monthly basis or prior to the commencement of a new treatment cycle. Instead of this intensive episodic education, a more frequent, bite-size coaching strategy will be adopted. Participants will receive daily push notifications from the educational library of CORA. This strategy is focused on empowering patients to be able to self-manage the recommended home care activities. The content of the messages include:

- general education to improve patients’ understanding about the disease process
- education for patients about their medications and associated side-effects
- information about the benefits of optimal adherence
- strategies to help prevent or delay side-effects
- education about coping and self-care skills
- psychosocial support
- information about safe handling, storage, and best practices in taking medications
- information about safety measures in disposing of bodily waste products and addressing other safety issues pertaining to the use of chemotherapeutic medications at home

#### Symptom Reporting and Management

Since patients have to take medications in the comfort of their homes without their oncology nurses or physicians to monitor or manage side effects, they need to be able to identify side effects early in order to institute management in a timely fashion. Symptom management is based on three premises that patients can:

1. Recognize symptoms at an early stage. Patients will receive educational messages, about common symptoms that accompany their OAMs such that they are able to identify these symptoms early and manage or report them in a timely fashion.
2. Assess severity of their symptoms. After patients have been able to identify their symptoms, they will be able to use the mobile app to assess the severity of their symptoms, after which they will be guided on appropriate management strategies.
3. Generate appropriate response to the symptom. Response to symptoms can be negative or positive. A common negative reaction is to ignore symptoms that may become self-perpetuating and eventually result in decreased medication adherence or increased toxicity. The positive response we aim to help patients develop is increasing competency at performing basic self-care activities so that in time, patients become experienced managers of these common symptoms during a repeat episode.

Existing symptom management protocols at the DFCI will serve as the framework of the symptom management algorithm. After symptoms have been identified and adequately quantified, the app will guide patients through self-care strategies to help them adequately manage the symptoms.

**Figure 2:**
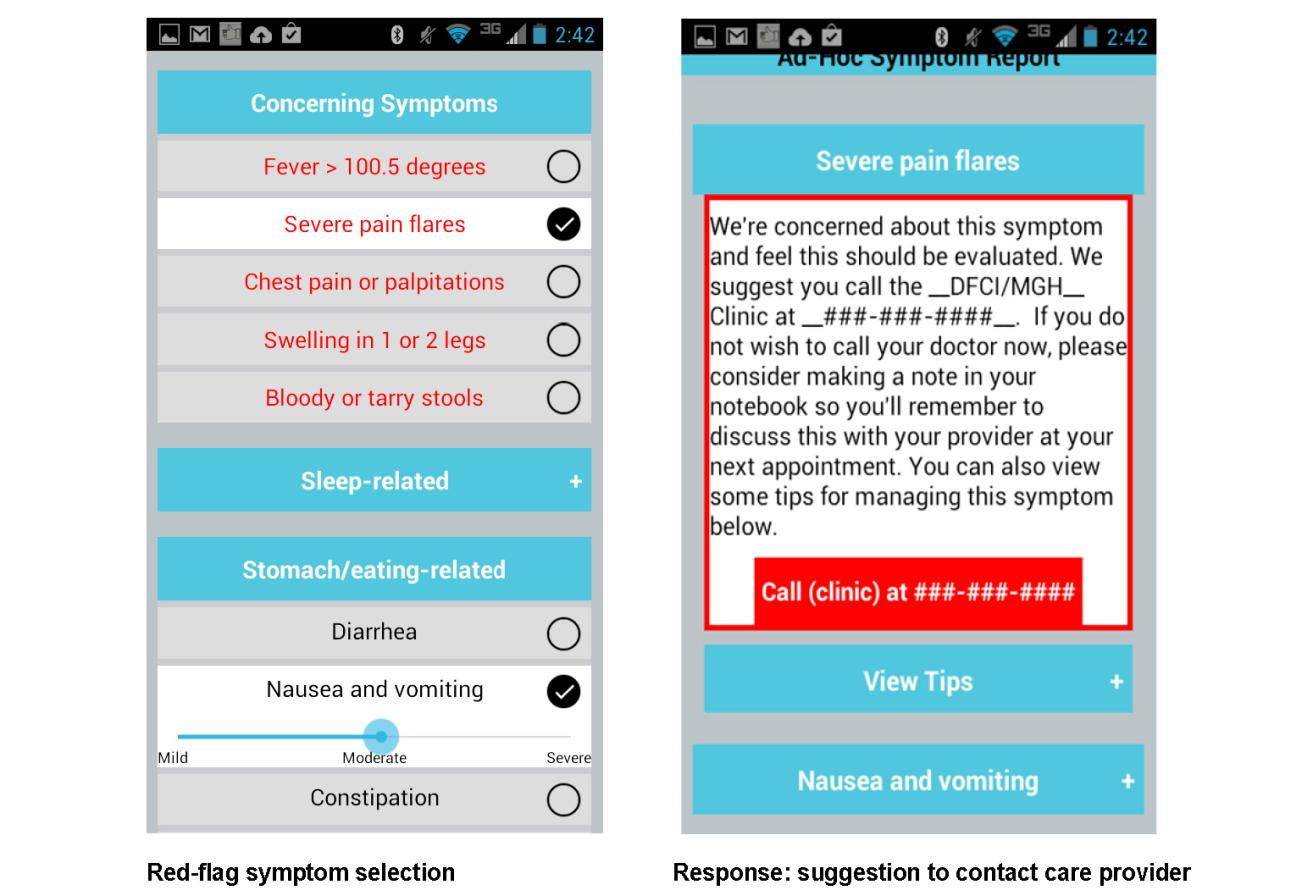
Red-flag symptom reporting.

**Figure 3:**
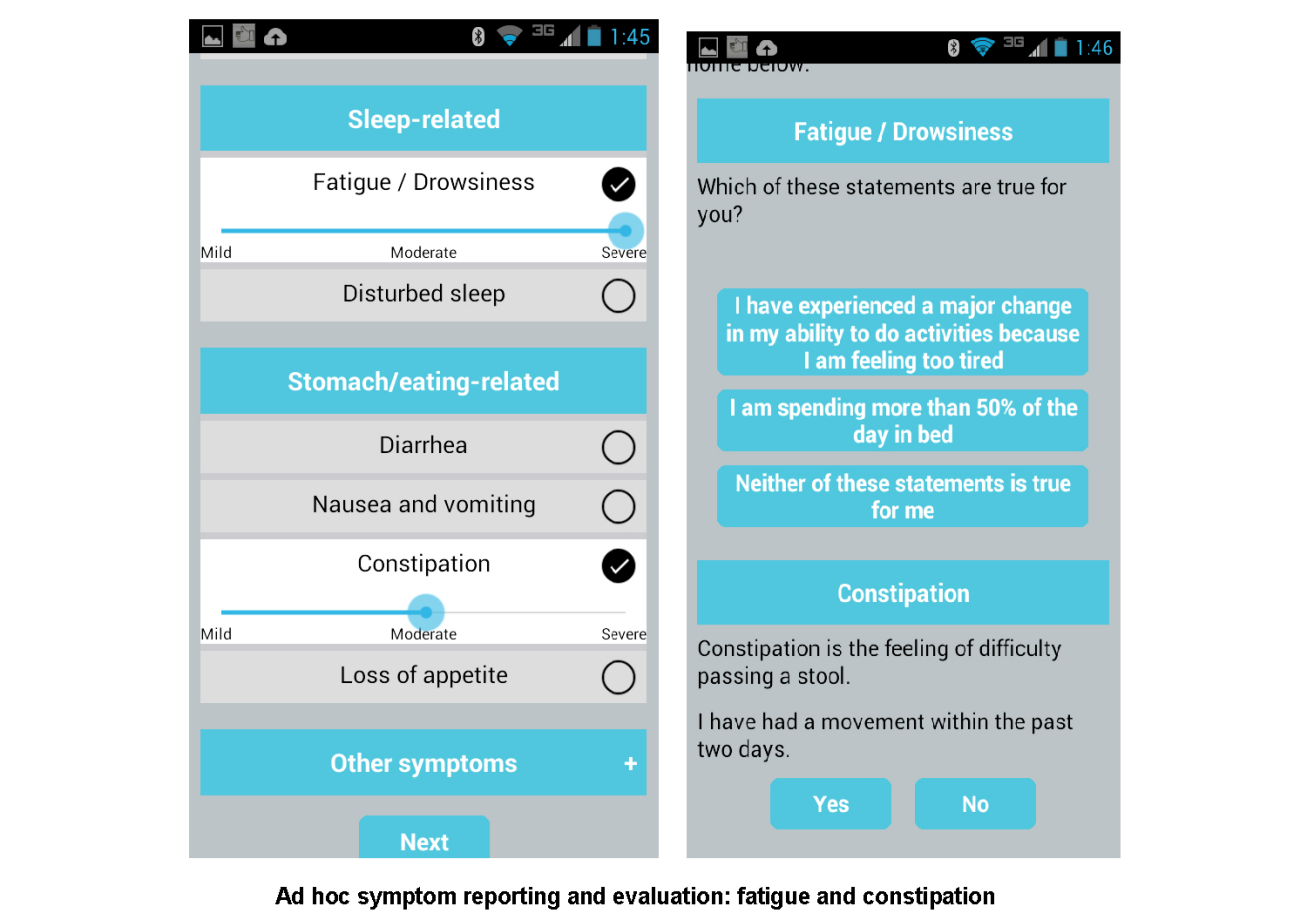
Ad hoc symptom reporting.

**Figure 4:**
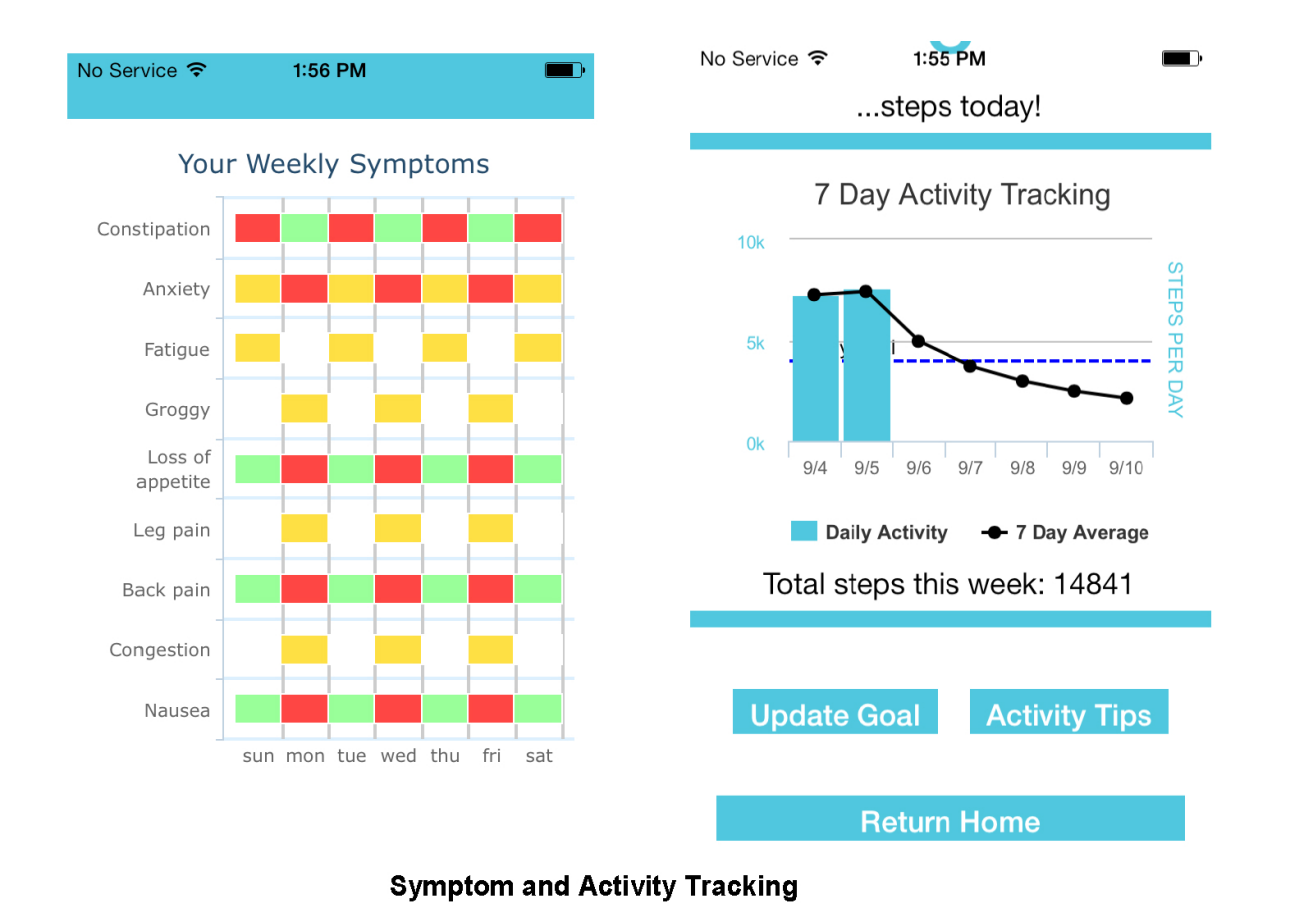
Symptoms and activity tracking.

Symptoms are categorized into red-flag symptoms (Figure 2) that notify the patients’ care providers and other symptoms that can be managed through the app. Participants will receive weekly prompts to report their symptoms, and the app will guide them in managing the identified symptoms. They will also have access to ad hoc symptom reporting (Figure 3) and the ability to track symptom progression (Figure 4). Screenshots of these functionalities are shown in Figures 2-4.

#### My Treatment Plan

Another key function of CORA is the ability of patients to create a treatment plan schedule at enrollment. They will be able enter basic information about their medications and personal preferences like drug names, dosages, scheduled breaks between cycles, food allergies and preferences, etc. They will also be able enter information about other supportive medications like anti-emetics or analgesics. CORA will use all the information entered to help personalize treatment for each participant. For example, a patient on a medication that is required to be taken on an empty stomach will receive a reminder to fast in preparation for the upcoming dose.

Other functionalities include a diary (a notepad integrated in the app allowing patients to log their symptoms or note questions they might have for their care providers at the next hospital visit) and activity tracking (participants will be able to monitor their step counts by using an activity tracker, Fitbit Flex, integrated into the mobile app). We hope that tracking both their activity levels and how they are feeling in general could better help them plan and cope more effectively with the disease.

#### Treatment Assignments

##### Intervention Group (CORA + Usual Care)

Subjects assigned to the intervention arm will continue to receive the standard OAMs care at DFCI as usual. In addition to usual care, they will also be able to use the CORA mobile app to manage their medications on their mobile phones for a duration of 3 months.

##### Control Group

Subjects assigned to the control arm will continue to receive only the standard OAMs care at DFCI as usual.

#### Randomization and Blinding

A computer program will be used to randomize subjects into the intervention or control arm in a ratio of 1:1 using random permutated blocks to optimize balance in each treatment at any given point in time during the study. Treatment assignment will be concealed in sealed envelopes prepared by a third party not directly involved in the study. Due to the nature of the intervention, it is difficult to blind the subjects to the treatment assignment, but it will be concealed from the investigators and the data analyst. In addition, the study investigators will not be directly involved in the day-to-day running of the study. This will be done by trained research assistants and a project manager who will report regularly to the investigators on the progress of the study.

### Outcome Measures

#### Data Collection Materials

One primary outcome and several secondary outcomes will be assessed in this trial. A number of data collection materials, including validated and study-specific questionnaires, will be used to assess study outcomes at multiple time points over the 3-month period. These tools will be completed in-person under the supervision of the research assistants during the study visits. Among these are the enrollment survey designed by investigators to collect demographic information and subjects’ technology use information, and the Patient-Health Questionnaire (PHQ-8) [18], which will be used as a screener for depression, a potential confounder in this study. If a subject is assessed to have severe depression on this tool, study staff will note this to the file and report immediately to the principal investigator who will duly notify the subject’s oncologist by phone. Additionally, such subjects will be encouraged to contact their physicians regarding this screen.

#### Primary Outcome

The primary outcome measure for this study is medication adherence, defined as the percentage of prescribed doses taken as captured by the MEMS device, which all study participants will receive at enrollment. The MEMS, like other measures of adherence, is not without its drawbacks, but it provides an objective measure of when the pill bottle was opened, which correlates well with the timing of medication self-administration. Currently, there is no gold standard measure of medication adherence, but the MEMS has been used extensively in medication adherence studies [19,20]. Additionally, we will also use a patient self-report tool, the Morisky Medication Adherence Scale (MMAS-8) to measure medication adherence [21]. This is an 8-item self-reported questionnaire used to measure medication adherence. Research has shown that self-reporting of medication adherence captures patient adherence to a reasonably accurate degree. The MMAS has been tested in several different settings and has been shown to demonstrate both concurrent and predictive validity of medication adherence. This survey will be administered in-person by trained research staff at study entry, midpoint, and at the end of the study.

#### Secondary Outcomes

The following secondary outcomes will be measured at various time points during the study:

- Symptom severity: This will be measured by the MD Anderson Symptom Inventory (MDASI), which is a validated and widely used survey in clinical practice and research [22]. It is used to assess multiple symptoms experienced by cancer patients and how the symptoms interfere with daily living. Its 13 core items include the most frequently occurring symptoms seen in various cancer types and treatment modalities. It will be administered in-person at study entry, midpoint, and end of study. However, because the tool also captures some symptoms that may occur and resolve acutely, we will also assess symptoms in between study visits at weeks 3 and 9. These in-between study assessments will be self-administered via REDCap, a secure, Web-based app for data collection customizable for individual research studies. It is free and complies with all Health Insurance Portability and Accountability Act regulations (HIPAA). It was developed by a multi-institutional consortium initiated at Vanderbilt University. Participants will receive the REDCap links in their in email to complete the surveys online.
- Hospital utilizations: This will be measured at the end of the study by a review of the electronic medical records for emergency department or urgent care clinic visits and in-patient admissions.
- Quality of life: This will be measured longitudinally by the Functional Assessment of Cancer Therapy-General (FACT-G) questionnaire administered in-person at study entry, midpoint, and end of study [23]. Now in its fourth version, this questionnaire is well validated and has been translated into nearly 50 different languages and has been used extensively worldwide. It has four subscales: Physical Well-Being, Social/Family Well-Being, Emotional Well-Being, and Functional Well-Being.
- Cancer-related fatigue: This will be assessed by the Functional Assessment of Chronic Illness Therapy - Fatigue Version 4 (FACIT-F) at study entry, midpoint, and end of study [24]. It is a self-administered 13-item questionnaire validated for use in chronic illness and frequently used with cancer patients.
- Anxiety: The Generalized Anxiety Disorder 7-Item Scale (GAD-7) will be used to assess anxiety [25]. This self-administered 7-item questionnaire is a valid and efficient tool commonly used in both clinical practice and research studies to assess the severity of anxiety.
- Usability and Satisfaction: This is a study-specific questionnaire designed by the authors to collect usability and satisfaction information.

### Statistical Analysis Plan

#### Sample Size Estimation

A sample size of 82 subjects, 41 in each arm (control vs intervention) is sufficient to detect a 10-point difference in mean medication adherence rates (measured by MEMS) between the two groups, assuming equal standard deviation of 16 using a two-tailed *t* test of difference between means with 80% power and a 2-sided alpha of .05. Considering a dropout rate of 20%, the sample size required is 104 (52 subjects per group). Medication adherence rates in the literature vary based on the methods of measurement. The assumptions used in our sample size calculations are based on similar studies, especially those that used the MEMs device as the primary measure of medication adherence [4,6]. A total of 52 participants will be randomly assigned to receive the mobile intervention and will also continue to receive the standard of care for OAMs at the DFCI (intervention group), while the remaining 52 participants will be assigned to the control group that will continue to receive the standard of care at DFCI (usual care group). Participants will be followed up for a total of 3 months.

#### Statistical Analysis

Data analyses will be done with data analysis and statistical software STATA, version 13, with an alpha of .05 set a priori. Although there are multiple assessment points, all subjects will be followed up for a total of 12 weeks in this 2-parallel group study design. The intention-to-treat approach will be used for all analysis. Descriptive statistics, means (normally distributed) and medians (skewed) continuous data and percentages for categorical variables, will be used to summarize baseline demographic and technology use characteristics by study arm. We will examine for group differences in the primary outcome, percentage of pills taken, by *t* tests or by the non-parametric Mann-Whitney tests if the data are not normally distributed. Logistic regression will be used to identify potential predictors of adherence. For secondary outcomes, continuous outcomes will be analyzed using *t* test or Mann-Whitney test, and categorical variables will be compared using chi-square tests. Given the longitudinal mode of data collection, a repeated measure analysis of variance will be used to evaluate changes from baseline.

### Ethics and Informed Consent

Procedures of our methods have been reviewed and approved by the Dana-Farber/Harvard Cancer Center Institutional Review Board (IRB), and the study will be registered on ClinicalTrials.gov. The app is secure and complies with all HIPPA requirements. Subjects will require a secure passcode to be able to access the app. All mobile phone numbers will be stored in a secure shared-drive available only to IRB-approved study staff. However, if any data breach or adverse effect occurs, the investigator will ensure that they are well documented and reported according to the IRB’s requirements, regardless of causality.

Participants will be sent a copy of the consent form in the mail along with the enrollment confirmation letter prior to the enrollment visit. They will be encouraged to review the study procedures and discuss their participation with their families. At the enrollment visit, subjects will be given the necessary time to review the consent form and will be encouraged to ask questions concerning their participation. The research assistant will go through the consent form with the subject to ensure their comprehension of study details. We will make it clear that their participation is completely voluntary and their decision to participate or not participate will not affect their management at DFCI. After the reviewing the consent form and reaffirming their interest in participating in the study, the subject will sign two copies of the consent form, one of which they will take home with them. All enrollment procedures, including the informed consent process, will be done by trained research assistants.

## Results

We expect to have results for this study before the end of 2016.

## Discussion

### Principal Considerations

Our focus on adherence to OAMs is timely because it is estimated that about 25% of cancer therapeutic drugs in development pipelines are oral medications [1]. Also, increased survival and requirement for chronic treatment for some of these cancers like chronic myeloid leukemia will continue to make adherence a prominent issue in the management of patients on OAMS. We hope that CORA can be used in a variety of settings to help patients better manage their medications and symptoms associated with the disease and or medications. Currently, there are only a few studies examining the problem of adherence to OAMs, and many of them are not randomized controlled trials. Although the sample size was small, the study by Simons et al investigating the effect of an intensified multidisciplinary pharmaceutical care program consisting of written and spoken information on adherence to capecitabine in patients with breast or colon cancer found that the mean daily adherence was significantly higher in the intervention group (96.8% vs 87.2%, *P*=.029) and that the intervention also prolonged the chances of patients still being treated with the medication at the end of the follow-up period (48% control group vs 83% in the intervention group; *P*=.019) [20].

Our study is one of the first clinical trials to use a mobile phone app to address the problem of adherence to OAMs. We chose to deploy the app on a smartphone because their adoption is rapidly increasing. Recent national data suggest that more than 56% of Americans now own specifically a smartphone [26]. Another major consideration for deploying this intervention as a mobile app is the additional privacy and security features that can be utilized on smartphones, which helped us to develop a more robust HIPAA-compliant intervention. Users would have to enter a passcode each time they logged on the app.

### Limitations

This trial will have limited generalizability of findings given that we are restricting our sample population to patients with renal and prostate cancer. However, CORA was designed for use in patients on any type of OAMs, so we believe that findings from this study could be generalized to other type of cancers. Another threat to generalizability is the fact that we limited study recruitment to one large academic medical center. We believe this should not be of too much concern because the app was designed so that it could easily be adapted for use in other settings.

### Conclusion

We have described the trial of a novel solution, deployed on a mobile phone app, for the emerging problem of adherence to OAMs. This innovative approach includes personalizing feedback and management based on patients’ own treatment regimen, baseline knowledge, and elucidated barriers to adherence, and holds great promise in improving overall adherence, safety, and clinical outcomes in these patients. We hope that future projects targeted at improving medication adherence in patients on OAMs can build on findings from this project.

## Abbreviations

CORA: ChemOtheRapy Assistant
DFCI: Dana Faber Cancer Institute
HIPAA: Health Insurance Portability and Accountability Act regulations
IRB: Institutional Review Board
MEMS: Medication Event Monitoring System
OAMs: oral anti-cancer medications
